# Heartburn in Staff of Golestan Medical University, Northeast of Iran

**DOI:** 10.4021/gr2009.06.1296

**Published:** 2009-05-20

**Authors:** Sima Besharat, Mahsa Besharat, Taghi Amiriani

**Affiliations:** a Golestan University of Medical Sciences, Golestan Research center of Gastroenterology and Hepatology, Iran

**Keywords:** Heartburn, BMI, GERD, Prevalence, Iran

## Abstract

**Background:**

Gastro-esophageal reflux disease (GERD) is the most common gastrointestinal disease in the west that has shown increasing incidence in Iran and Asian countries. The main presentations, described for GERD, are heartburn and acid regurgitation.

**Methods:**

In this cross-sectional study in 2006, all personnel of Golestan Medical University (Northeast of Iran) were enrolled. A questionnaire consisting of demographic data, symptoms and risk factors was completed for all volunteers. Height and weight were measured. Chi-square and Non-parametric tests were used for analysis.

**Results:**

Symptoms of heartburn were seen in 60% of all 155 studied subjects. No significant relationship was seen between symptoms and variables like age, gender, BMI and tribes. Symptoms were more common in married ones (P < 0.05).

**Conclusions:**

Heartburn prevalence was high in this study. Heartburn was seen more in women and in married. The probable underlying etiology and explanation for these results should be studied more.

## Introduction

Gastro-esophageal reflux disease (GERD) has been known for a long time, but clinical attention is drawn only during the recent years. Currently, it is one of the most common gastrointestinal problems in the West and increases in Iran and other eastern countries as well [1]. It may cause important complications including impaired quality of life, erosive esophagitis, esophageal ulcers and strictures, specialized columnar metaplasia (Barrett’s esophagus), dysplasia, and adenocarcinoma [2-10]. Impaired sanitary conditions and difficulty in accessing health care facilities are among the probable risk factors in developing countries [11].

GERD may manifest in different ways with heartburn being the most common symptom. The patient might regurgitate bitter material or burp it up into his mouth. Reflux can occur during the day or night. Many authors consider heartburn as an indicator of this disease [1-2]. It was reported that prevalence of heartburn can be related to being a single woman, having low educational level, insomnia, mental disorders and obesity [12-18]. However, reported symptoms may under-represent the true prevalence of the disease because mucosal damage is not considered [1].

GERD can result in disturbed quality of the life and some important and serious consequences like esophageal adenocarcinoma [19-22].

Few studies are done on the characteristics of the patients suffered from GERD, despite the increasing trend in the field of diagnosis and treatment [23]. The aim of this study was assessing heartburn prevalence rate in the staff of Golestan University of Medical Sciences (Northeast of Iran) and some of its associated factors.

## Methods

This study was done in 2006 in Golestan University of Medical Sciences (GOUMS), northeast of Iran.

We searched for a simple suitable questionnaire, and after revising and summarizing some available ones, we designed another questionnaire with 24-items including demographic data (age, gender, marital status, current job, etc); personal habits (smoking, tea consumption, etc); symptoms (heartburn, non-cardiac chest pain, food regurgitation and acid reflux); times of being symptomatic (at least once a week, once a week or more, intermittent/ occasionally); the duration between meals and sleeping; special foods which aggravate symptoms; endoscopic examination and seeking physician visits. Among these questions, 10 were specific to symptoms of GERD. It was a self-administered questionnaire. The research proposal was approved in the Ethical Committee of Golestan University of Medical Sciences.

A pilot study was done on 15 subjects; test-retest (at 2-3 week interval) was done for assessing reliability and Cohen's kappa coefficient was used for statistical analysis. All questions were considered understandable by the subjects, except for one. This was mentioned orally by the interviewers and corrected in the main questionnaire.

After it was shown that the questionnaire was reliable, it was administered to all staff of the main campus of GOUMS.

In the present population, individuals who complained of heartburn at least twice a week were considered as having GERD. Although this criterion is somehow uncertain [24], we assumed that the presence of heartburn 2 or more times a week is highly suggestive of the presence of GERD [25].

Height and weight of subjects were measured. Body mass index [BMI = weight (kg) / square of height (m^2^)] was calculated in each person. Those with a BMI lower than 20 kg/m^2^ were considered as thin; 20-25 kg/m^2^ as normal; 25-30 kg/m^2^ as overweight; and > 30 kg/m^2^ as obese [25].

Data was coded and analyzed with SPSS-13 statistical software. Chi-square and Fisher’s exact test were used to obtain the relationships. Non-parametric tests were used to compare means.

## Results

Of the 280 eligible staff in the main campus of the University, 155 (73 men, 47.1%) returned the completed questionnaire and were enrolled into the study (response rate = 55.4%). Mean age was 33.9 ± 8.2 years, mean weight was 69.5 ± 15.2 kg and mean BMI was 24.98 ± 4.34 kg/m^2^. These people reported to drink an average of about 4 cups of tea per day. Fars ethnicity was the most common (88.4%). Married people comprised 75.5%. Three (1.9%) were cigarette smokers; 5 (3.2%) used hookah (water pipe) and 2 (1.3%) used both.

Overall 93 people (58.1%) reported at least one episode of heartburn in a week and 10 (10.9%) of them had a history of endoscopic examination with normal report. Thirty- two (20.5%) reported more than one symptom, Tables 1 and 2.

**Table 1:** Type of symptoms in staff of Golestan University of Medical Sciences

Symptoms	Number	Percent
Asymptomatic	62	40.0
Heartburn	27	17.4
Acid reflux	24	15.5
Regurgitation	7	4.5
Chest pain (Non-cardiac)	3	1.9
More than one symptom	32	20.5
Total	155	100.0

**Table 2:** Frequency of symptoms

Times of being symptomatic	Number	Percent
At least once a week	8	9.4
once a week or more	10	11.8
Intermittent/ocassionaly	75	78.8
Total	93	100

Gender, BMI, age, weight, height, and ethnic background had no significant relationship with symptoms of heartburn, Table 3. Self-reported tea color was not significantly different between the two groups (symptomatic and non-symptomatic), Table 4.

**Table 3:** Comparison of two groups (symptomatic and asymptomatic) regarding different parameters

	Number	%	Gender				Age (years)		BMI (Kg/m^2^)	
			male		female		Mean	SD	Mean	SD
			No.	%	No.	%				
**Asymptomatic**	62	40	33	53.2	29	46.8	34.32	8.58	24.82	4.01
**Symptomatic (Heart burn, acid reflux)**	93	60	40	43	53	57	33.58	8.02	25.09	4.5
			No significant difference				No significant difference		No significant difference	

**Table 4:** Comparison of two groups (symptomatic and asymptomatic) regarding to the self-reported tea color

	Numbers	%	Tea color					
			Light		Mild		Dark	
			No.	%	No.	%	No.	%
**Asymptomatic**	62	40	9	14.8	49	80.3	3	4.9
**Symptomatic (Heart burn, acid reflux)**	93	60	23	25	63	68.5	6	6.5
			No significant difference					

Marital status and symptoms had significant relationship (P < 0.05). It means that married people had a relative risk of 1.5-fold to suffer from heartburn.

## Discussion

Gastro-esophageal reflux disease is one of the most common chronic diseases in adults in the US [13]. Prevalence of this disease in Western countries can not be the unique representative of its pattern in the world, more data from developing countries are needed. Currently, there are few prevalence data from developing or underdeveloped nations [11].

An approximate prevalence of 10-20% is reported for GERD, defined by at least weekly heartburn and/or acid regurgitation in the Western world while in Asia this was lower (less than 5%) [11].

The present study, clarified the prevalence and some characteristics of heartburn, which may, depending on the frequency and duration, be a presentation of GERD. The definition of GERD is not uniformly accepted and several definitions have been proposed [26, 27], all of them assume that patients who complain of heartburn, regardless of the presence of injured esophageal mucosa, have GERD [18], which is not always true.

In the present population, individuals who complained of heartburn at least twice a week were considered as having GERD. Although this criterion is somehow arbitrary [23], we assumed that the presence of heartburn 2 or more times a week is highly suggestive of the presence of GERD [24]. Present data showed a prevalence of 58% for heartburn. In 9.4% of participants, heartburn was seen at least once a week.

It was reported very lower in Tabriz (2000), about 2.7% in a general population. In 80% of patients, special food worsened symptoms of heartburn and the most common associated sign was sialorrhea [28], this is similar to our finding to some extent.

Ehsani Ardakani et al reported a 39.7% prevalence of gastro-esophageal reflux in Tehran (1999) and its prevalence in cigarette smokers was higher than others [29]. Cigarette smoking and GERD symptoms had no significant relationship in our study. It can be explained best as cigarette smokers were infrequent (or under-reported) in our study.

In other studies, prevalence of heartburn was 6.2-40%, and heartburn once a week at least was seen in 4.6%. BMI higher than 25 kg/m^2^ was the only dependent variant [13-15, 30].

Studies from East and South-East Asia have shown much lower prevalence rates [17] and it is generally believed that the disease is not as frequent in the East [30, 31]. Recent reports have challenged this concept [10].

In our investigation, heartburn was seen more in women but no significant differences were seen. Nevertheless, there is no evidence that women respond to anti-secretary therapy or anti-reflux surgery any differently than men do, due to the effect of sexual hormones [32]. It was suggested that the prevalence of acid reflux episodes might be slightly higher in men, whether normal or symptomatic, however, this does not appear to be of clinical importance [33]. Maybe, women predominance is related to higher reporting and sensitivity of them, it needs more evaluation.

In a Brazilian study, average age in two genders was equal and women were more affected, like our findings, and there was seen that GERD prevalence was higher in the older people. BMI was the same between groups and in normal range [13].

In China, prevalence rate in two genders has no significant differences. GERD symptoms were more seen in divorced and detached people or who had a hard work [29]. We found that married people reported symptoms of heartburn more than singles.

It has been known that overweight and obese persons are at increased risk for GERD [33]. We could not confirm relationship between BMI, age, weight and height, ethnic and existence of symptoms from statistical point of view. Most of the people in our inquiry (affected and healthy), were in the normal range.

In De Oliveira’s report, heartburn prevalence was higher in women, singles, low educational levels, insomnia, mental disorders and obesity. It was seen that sex and anthropometric variables and mental characteristics were the main factors [12].

Other studies reported that positive family history, living in south Asia, cigarette smoking, alcohol consumption and some special medicines are related to GERD symptoms [16-17].

It seems that almost all risk factors which are suggested for heartburn and GERD are modifiable and probably can be corrected with changing the life style to prevent the next coming reflux complications. Prompt diagnosis and adequate maintenance therapy are the cornerstones of appropriate management of these patients. Dietary and lifestyle changes contribute significantly to this new epidemic and should be addressed properly in each counseling occasion with patients and their families.

As there is no gold standard for diagnosis of GERD, except of careful history taking, epidemiologic studies may be challenging. Global consensus on a symptom-based definition of GERD is necessary.

This study had some limitations. First of all, it was done in a subgroup that is more alert about the symptoms of disease and seeks treatment considerations earlier. Secondly, occasional reflux symptoms are not considered as a disease and may not even be a useful risk factor for any significant esophageal pathology, except when endoscopic examination would be performed and findings suggest the underlying disorders. Thus a true definition and clear cut-off is needed to better clarify the symptoms related to the probable pathology.
